# Angiogenesis-Related Biomarkers (sFlt-1/PLGF) in the Prediction and Diagnosis of Placental Dysfunction: An Approach for Clinical Integration

**DOI:** 10.3390/ijms160819009

**Published:** 2015-08-13

**Authors:** Ignacio Herraiz, Elisa Simón, Paula Isabel Gómez-Arriaga, José Manuel Martínez-Moratalla, Antonio García-Burguillo, Elena Ana López Jiménez, Alberto Galindo

**Affiliations:** 1 Fetal Medicine Unit-Maternal and Child Health and Development Network (SAMID), Department of Obstetrics and Gynaecology, Hospital Universitario “12 de Octubre”, Universidad Complutense de Madrid, 28041 Madrid, Spain; E-Mails: iherraizg@gmail.com (I.H.); elisa.simon87@gmail.com (E.S.); paulagomezarriaga@hotmail.com (P.I.G.-A.); josemoratalla@hotmail.com (J.M.M.-M.); agarciaburguillo@salud.madrid.org (A.G.-B.); 2 Department of Clinical Biochemistry, Hospital Universitario “12 de Octubre”, Universidad Complutense de Madrid, 28041 Madrid, Spain; E-Mail: annaelenalopp@yahoo.es

**Keywords:** placental insufficiency, preeclampsia, intrauterine growth restriction, placental abruption, angiogenesis, sFlt-1, PlGF

## Abstract

Placental dysfunction is involved in a group of obstetrical conditions including preeclampsia, intrauterine growth restriction, and placental abruption. Their timely and accurate recognition is often a challenge since diagnostic criteria are still based on nonspecific signs and symptoms. The discovering of the role of angiogenic-related factors (sFlt-1/PlGF) in the underlying pathophysiology of placental dysfunction, taking into account that angiogenesis-related biomarkers are not specific to any particular placental insufficiency-related disease, has marked an important step for improving their early diagnosis and prognosis assessment. However, sFlt-1/PlGF has not been yet established as a part of most guidelines. We will review the current evidence on the clinical utility of sFlt-1/PlGF and propose a new protocol for its clinical integration.

## 1. Introduction

Placental dysfunction (PD) is involved in a spectrum of obstetrical conditions including preeclampsia (PE), intrauterine growth restriction (IUGR) and placental abruption (PA) which remains as worldwide major cause of morbidity and mortality in both the mother and the fetus [1]. In the last years, a wide body of evidence supports that the state of placental insufficiency triggers an imbalance in the placental release of angiogenesis regulatory factors to the maternal circulation, characterized by elevated concentrations of anti-angiogenic factors such as soluble fms-like tyrosine kinase 1 (sFlt-1, also referred to as sVEGFR-1), and decreased concentrations of pro-angiogenic factors such as placental growth factor (PlGF) [2], which are directly involved in a substantial proportion of the clinical manifestations associated to PD, especially in the spectrum of the most severe and precocious forms.

Despite that our understanding of these processes has increased strikingly, our ability to manage these diseases has not improved accordingly. Proof of this is the fact that their diagnostic criteria are still based on unspecific clinical, ultrasound and laboratory findings, rather than on their pathogenic origin, and such diagnostic criteria have scarcely been modified over the past decades. As a paradigm, the current gold standard of PE diagnosis relies on the demonstration of new-onset of hypertension and proteinuria in the second half of pregnancy, whose presence does not always precede the onset of complications and has no predictive ability of PE-related adverse outcomes [3]. In the case of IUGR, ultrasound findings—mainly estimated fetal weight and Doppler hemodynamics—approach the diagnosis but, not infrequently, the differentiation between IUGR and constitutionally small fetuses is extremely difficult, even in expert hands [4]. PA, defined as the complete or partial separation of the placenta prior to delivery, usually remains unrecognized until symptoms, such as vaginal bleeding and abdominal pain, occurs, when fetal wellbeing is already severely compromised [5]. Therefore, these diagnostic limitations may lead to recognize PD in an advanced stage of the disease, hindering its early diagnosis, its optimal management, and potentially leading to severe complications for both the mother and the fetus. That is why current efforts are focused on implementing strategies in the clinical setting based on the use of *in vitro* diagnostic tests for PD, especially the sFlt-1/PlGF ratio, in order to improve the prediction and early diagnosis of the PD.

The aim of this article is to review the current evidence on the clinical utility of the sFlt-1/PlGF ratio at different points in pregnancy and, accordingly, make a proposal for its clinical implementation. This could allow predicting women at risk of PD, to make an early and reliable diagnosis, to provide information about the state of the disease for better selecting the time for intervention, and, in short, improving maternal and perinatal care in PD affected pregnancies.

## 2. Angiogenesis-Related Biomarkers of Placental Dysfunction in Physiological and Pathological Conditions

Although the underlying origin of PD has not been fully elucidated, it is commonly accepted that PD-related diseases comprise a spectrum of obstetrical syndromes that are characterized by a defective deep trophoblastic invasion and impaired maternal spiral artery remodeling in the first half of pregnancy, leading to inadequate placental perfusion in the second half. The period of trophoblast invasion that takes place between 12–14 and 20–24 weeks is the main determinant of the quality of placental implantation, since this is when maternal myometrial spiral arteries are transformed into low-resistance and high-flow vessels. Such phenomena are necessary to deliver an adequate blood flow to the intervillous space, in which the materno-fetal exchange takes place. It has been hypothesized that reduced remodeling of the spiral arteries in their myometrial segments precludes the proper regulation of the flow pressure to the intervillous space, since the arterial contractile properties remain. This leads to the appearance of hypoperfusion-reperfusion phenomena which in turn causes damage in the villous architecture, and an impairment of the materno-fetal exchange finally causing IUGR [6]. Occasionally, this shallow implantation can also favor the placental detachment, leading to acute or chronic PA. Finally, the maternal endothelial damage is believed to be mediated by the excessive release of toxins from the dysfunctional placenta to the maternal circulation that cause peripheral vasoconstriction, in an attempt to raise maternal blood pressure and thus increasing the oxygenated maternal blood flow through the intervillous space, but eventually generating a systemic vascular disorder known as PE [7].

Over a decade ago, the plausible identity of the circulating toxin released by the insufficient placenta that appears to be an essential contributor to the mechanisms of maternal endothelial dysfunction, was recognized as sFlt-1, an anti-angiogenic protein of placental origin that is key to the regulation of angiogenic homeostasis during pregnancy [8]. This has been shown to be markedly elevated in many cases of PD, especially in those that appear early in pregnancy. sFlt-1 is a soluble truncated variant of the Flt-1 receptor which lacks the cytoplasmic and transmembrane domain, but conserves the ligand-binding domain. Flt-1 (or VEGFR-1) is a vascular endothelial growth factors family receptor present on vascular endothelial cell membranes, which is also expressed in the placenta, mainly by the syncytiotrophoblast. Flt-1, as well as kinase-insert domain containing receptor (KDR or VEGFR-2), have high affinity for the vascular endothelial growth factor-A (VEGF-A), which is the essential factor involved in placental vascular development, proliferation and survival of endothelial cells, vascular permeability, and fenestration of endothelial cells. For example, VEGF-A is secreted in the kidney by podocytes to maintain the glomerular endothelial cells in a healthy state, and its suppression results in severe proteinuria. PlGF is also a VEGF family member with pro-angiogenic activity and abundantly expressed in the placenta, which binds to Flt-1, but not KDR, and acts by enhancing the action of VEGF-A [9]. In response to hypoxia, alternative splicing of the Flt-1 gene is generated in the form of sFlt-1 mRNA, and sFlt-1 is produced and secreted from the placenta to the maternal circulation, causing a reduction of the bioavailability of the pro-angiogenic factors VEGF and PLGF by binding to them. This mechanism of action is similar to cancer therapies using VEGF-neutralizing antibody to reduce tumor angiogenesis, and whose most common side effects are hypertension and proteinuria. Furthermore, elevated levels of sFlt-1 in pregnant animal models promote hypertension and development of the typical renal features seen in PE, such as proteinuria and glomerular endotheliosis, what is consistent with the existing evidence in humans of its critical role in the pathophysiology of PE [10].

Nevertheless, the anti-angiogenic state does not always result in the development of the maternal syndrome of PE. The reasons for this are still unclear, but it is possible that PE develops once an individual threshold of angiogenic imbalance is reached. A combination of sufficiently large and prolonged endothelial insults, together with a constitutional maternal sensitivity to its effect, is required. Thus, the most seriously dysfunctional placentas produce extremely high amounts of sFlt-1 from the earliest stages of pregnancy, leading to early-onset PD in virtually all cases. Furthermore, the greater the maternal sensitivity to endothelial damage, the earlier and more severe the PD. On this part of the spectrum, the coexistence of PE and IUGR is frequent [11]. PA, although a rare event, also shows association along with preceding alterations in angiogenesis-related biomarkers [12], and very high values of the sFlt-1/PlGF ratio have been described shortly before the onset of PA [13,14]. Lesser degrees of placental insufficiency and anti-angiogenic insult might result in variable forms of PD depending on the maternal constitution. Late-onset DP is more commonly associated with less or no placental damage in women with long-term cardiovascular risk factors, such as chronic hypertension, renal disease, thrombophilia, diabetes and obesity, in which even the physiological changes at the end of pregnancy can induce PD [15]. Late-onset PE usually presents with adequate or large for gestational age fetuses, low rates of severe complications, and normal angiogenic profile, although there is a subtype of late-onset PD with altered angiogenic profile that is prone to IUGR [16] and adverse outcomes [17]. Finally, a certain degree of failed placentation has been also found in a low proportion of full-term normal pregnancies of not susceptible women [18,19]. According to a suggestive hypothesis, all pregnancies are intended to suffer from PE before or after, and whether they do so or not depends on competition between the respective timing of delivery and of PE [20]. This increased susceptibility to develop PE as the pregnancy progresses, even in the absence of defective deep placentation, has been explained by the appearance of alternative mechanisms that may reduce placental perfusion at this stage. Among them, an increased terminal villous volume that constrict intervillous spaces, superimposed obstructive lesions of the spiral arteries such us acute atherosis and thrombosis, and increased uterine contractility have been involved [21]. A role for the growing fetal demands for nutrients that may overcome the placental offer has also been suggested [22]. Ultimately, in the late pregnancy, the limits between normal and dysfunctional placenta are blurred.

### Clinical Keys for Interpreting Angiogenesis-Related Biomarkers in Pregnancy: The sFlt-1/PlGF Ratio

**Figure 1:**
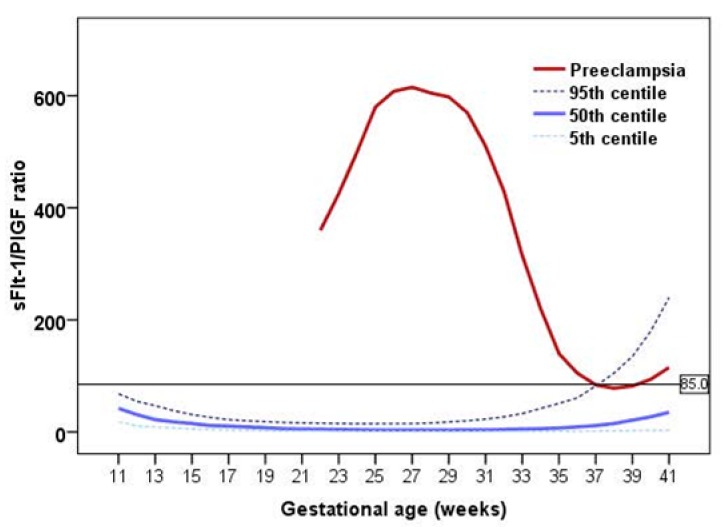
Maternal serum concentrations of the sFlt-1/PlGF ratio throughout pregnancy: reference normal values (5th/50th/95th centiles) are represented in blue and mean values at time of diagnosis of preeclampsia are represented in dark red. Maximum differences between cases and normal pregnancies occur during the early phase, especially at 24–28 weeks. In the late phase, and especially in term pregnancies, considerably overlapping is observed. For visualization, the sFlt-1/PlGF ratio cutoff of 85 is shown as a black horizontal line. Data have been extracted from references 24 and 39.

During normal pregnancy, sFlt-1 levels are stable until 29–33 weeks, when they rise steadily until delivery. On the other hand, levels of PlGF begin to rise progressively from the first trimester and more rapidly in the second trimester, reaching its acme around 29–33 weeks, and decline thereafter. In PD, maternal circulating sFlt-1 levels are significantly increased more than one month before the onset of the early detectable clinical symptoms [23]. In the case of PlGF, significant lower concentrations in women who later develop PD are seen from the end of the first trimester. The evolution of the sFlt-1/PlGF ratio throughout pregnancy is depicted in Figure 1. In general, early-onset PD is more readily detectable than late-onset PD by measuring the angiogenic imbalance, since the former show more altered values, with little overlap with normal ongoing pregnancies at the same gestational age. Conversely, with advancing gestation, sFlt-1 and PlGF values in normal and PD tend to converge, and it becomes more difficult to discern between them [24]. Furthermore, angiogenesis-related biomarkers are informative of the degree of PD, but are not specific to any particular PD-related disease.

## 3. From Bench to Bedside: Rationale for the Clinical Implementation of the sFlt-1/PLGF Ratio

Discovering the role of angiogenesis-related biomarkers in pregnancy, and especially in PD, has opened an exciting door to an etiologic (not symptomatic) approach of these pathologies that may challenge their current prediction, diagnosis, and even treatment, in the near future (Table 1). However, there is still limited experience on daily practice, and so there are still few clinical guidelines supporting its inclusion. Despite this, many efforts are been made to change this situation, and some applications of the angiogenesis-related biomarkers are already available. Special mention deserves the creation of a first-trimester risk calculator for PE, freely available online (https://courses.fetalmedicine.com/calculator/pe?locale=en), using a combination of markers among which is PlGF. As discussed later, the routine implementation of such a calculator is still under controversial discussion, since high-quality evidence about its benefits in terms of cost reduction and improved outcomes are limited. Regarding the second half of pregnancy, an initial step towards the routine use of the sFlt-1/PlGF ratio was given in 2010, when the first automated test for the measurement of the sFlt-1/PlGF ratio was approved for clinical use in Europe [25]. Recently, additional steps have been made with the inclusion of the sFlt-1/PlGF ratio in the German guidelines on PE as an “aid in diagnosis” biomarker, and the publication of a consensus statement on the use of the sFlt-1/PlGF ratio, in which experts in the field provide specific statements regarding “when?” and “in which women?” should the sFlt-1/PlGF ratio be measured and “what should be done with the results?” according to current knowledge [26]. Hereafter, we will review the evidence supporting the potential clinical implementations of the angiogenic-related biomarkers in four distinct stages of PD development.

**Table 1:** Potential clinical applications of the angiogenesis-related biomarkers (sFlt-1/PlGF ratio) in placental dysfunction-related disorders.

Proposed Application	Comments
Prediction of PD and stratification of care	→First trimester (11–14 weeks): for the selection of women potentially benefit from the use of preventive measures such as low dose aspirin (only PlGF); →Second and third trimester: for the precise stratification of patients that should receive intensive following-up in a specialized center.
Early diagnosis of PD	sFlt-1/PlGF ratio increases significantly 5 to 8 weeks before the appearance of clinical manifestations.
Differential diagnosis	→Uncertain cases; →Atypical cases.
Management of PE	Categorize those cases susceptible to receive an expectant management.
Cost-efficiency	Avoidance of other diagnostic tests or reducing admissions for false PD suspicion.
Treatment	Development of new treatments based on the angiogenic balance regulation and monitorization of its efficacy (especially sFlt-1).

PD, placental dysfunction; PE, preeclampsia; sFlt-1, soluble fms-like tyrosine kinase1; PlGF, placental growth factor.

### 3.1. First-Trimester Prediction and Prevention of Placental Dysfunction-Related Disorders

Low-dose aspirin prophylaxis of PD has been advocated under the hypothesis that its antiaggregatory and vasodilatory properties may promote a greater depth of placental invasion. In the last 30 years, more than 60 randomized trials have been conducted to confirm the effect of aspirin on the incidence of PD. Disappointingly, many of them, including those of larger size [27,28,29], failed to demonstrate such preventive usefulness. However, most pregnant women in these studies started taking aspirin after completion of the trophoblast invasion process. Subgroup meta-analysis of these studies suggested that low-dose aspirin: (1) should be taken before 16 weeks of pregnancy, when the trophoblast invasion is likely to be modified; (2) such strategy only benefits woman at high-risk for defective placentation; and (3) achieves its maximum preventive effect on the more severe and earlier cases. Lessons from low-dose aspirin can probably be extrapolated to other promising preventive treatments under investigation, such as dietary interventions, pravastatine, heparine, metformin or antihypertensives.

These observations have led to the interest in identifying pregnancies likely to have an impaired placentation early, by the end of the first trimester. Indeed, assessment of risk factors for PD already present in the maternal history should be part of the routine booking antenatal visit. Thus, the National Institute for Clinical Excellence (NICE) promotes the first-trimester identification of high-risk and moderate-risk factors and advise women to take low-dose aspirin prophylaxis from 12 weeks of pregnancy until birth if at least two moderate-risk or one high-risk factors for PD are present [30]. The main predisposing variables in the first-trimester screening for PD are listed in Table 2. However, the performance of this screening is suboptimal, yielding a detection rate for PE of 34.6% with a false-positive rate of 10.6% [20]. Uterine artery Doppler and biochemical markers alone also perform modestly at this stage of pregnancy, when the pathogenesis of PD is still incompletely developed. Regarding angiogenic-related biomarkers, significantly lower PlGF serum concentration in women who lately developed early and late-onset PE compared to the controls have been consistently found, having become an undeniable marker for the first trimester. However, concerning sFlt-1 and sFlt-1/PlGF ratio, studies on its effectiveness as first trimester predictive markers have yielded contradictory results [31,32,33].

**Table 2:** Identifiable risk factors used for combined placental dysfunction-related conditions screening at the end of the first trimester *.

Maternal History		Physical Examination	Ultrasound Findings	Serum Biomarkers
High-Risk Factors	Moderate-Risk Factors			
Previous PD related disorder †	Maternal age	Mean arterial BP	UA-mPI	PlGF
Chronic HT	Parity	Weight		PAPP-A
Chronic kidney disease	Familiar history of PD related disorder †	Height		
Diabetes Mellitus	Ethnicity			
Trombophilia, SLE	Method of conception			

BMI, body mass index; BP, blood pressure; HT, hypertension; PAPP-A, pregnancy-associated plasma protein A; PD, placental dysfunction; PlGF, placental growth factor; SLE, systemic lupus erythematosus; UtA-mPI, uterine artery mean pulsatility index. * This strategy is still under investigation and its routine use cannot be recommended until new high-quality studies demonstrate its cost-effectiveness and its efficacy for improving outcomes; † includes preeclampsia, intrauterine growth restriction and placental abruption.

Following the successful example of first trimester screening for common aneuploidies, the way to improve the predictive value in the first trimester has been found by moving towards a model of combined tests. Using such an approach in different combinations at 11+0–13+6 weeks of gestation, a sensitivity and specificity of about 90%–95% for early-onset PE has been reported [34]. Moreover, PD-related disorders other than PE benefits from this first-trimester screening [35]. However, not all groups have been able to reproduce these excellent results [36].

At this point, the question remains if first-trimester screening for PD is a real useful and cost-effective tool for changing the natural course of pregnancy. From the payer perspective, a cost-effectiveness model of this intervention was favorable for cost-saving in an Israeli setting [37], but this has not been ratified by a model validation in other healthcare systems. The usefulness of low-dose aspirin—or any other given prophylactic measure—to improve maternal or perinatal outcomes in those women who have a positive first-trimester combined screening for PD is still under debate. Promising results have been recently reported, but randomized clinical trials, which are now underway, will have to confirm them. If so, we must be aware that a universal first-trimester prediction and prevention strategy for PD should be promptly incorporated into clinical practice [38].

### 3.2. Second Trimester (24–28 Weeks): Stratification of Care in Asymptomatic Patients

Regardless of whether the preventive effect of aspirin is greater or smaller, pregnancies recognized at high-risk for PD should be intensively followed. We propose the gestational age window of 24–28 weeks as a starting point, mostly around 26 weeks, and the rationale for this approach is as follows:

(1) *Pathophysiological*: at this stage of pregnancy, the trophoblast invasion is completed, and the conversion of spiral arteries has been definitively established. It is usually from this point that the placenta must prove that is suitable to supply the growing fetal requirements or, otherwise, clinical manifestations of PD may begin to be apparent. Not by chance, great differences (with almost no overlap) in the sFlt-1/PlGF ratio between early-onset PD cases and normal pregnancies begin to be appreciated in this period [23,39].

(2) *Optimal performance of predictive markers*: direct knowledge of placental histopathology is not possible for the clinician while pregnancy is ongoing. However, it is possible to obtain surrogate information about the status of the uterine vasculature by means of non-invasive Doppler study of the uterine artery (UtA) flow resistance [40]. This simple and widely available test can be carried out at any time during pregnancy, although its performance improves as the trophoblastic invasion progresses, since it better reflects the definitive state of placental implantation [41]. While UtA Doppler at 11–14 weeks has a sensitivity of about 40%–50% for a specificity of 90%–95% for early-onset/severe PD, with a positive likelihood ratio of approximately 5–6, the diagnostic accuracy at 18–22 weeks improves to about 75%–80%, 90%–95%, and 10–15, respectively [42,43,44]. Persistently-abnormal UtA resistances at 26–28 weeks further increase the risk, while normalization at this stage is associated with similar results to the general population [45]. However, routine screening of PD by means of UtA Doppler is not currently recommended due to its very low positive predictive values (<15%), and the poor results seen in high-risk women, showing that impaired placentation is not the only substrate of PD.

The measurement of the angiogenesis-related biomarkers at the end of the second trimester, at around 26 weeks, in pregnancies with persistent abnormal UtA Doppler has shown to overcome this limitation, substantially improving the diagnostic accuracy. This strategy provides 80%–100% sensitivity with 89%–100% specificity for identifying early-onset PE and/or IUGR [46,47,48], and 85.7% sensitivity with 83.7% specificity for the need of inducing delivery before 34 weeks [49]. Most importantly, positive predictive values are >50%, which provides much better information for the stratification of care. This early recognition of highly likely PD at a “pre-symptomatic stage” using angiogenic factors may improve therefore the management and, as a consequence, the prognosis of affected pregnancies. These women could be involved in their surveillance by being aware of every specific sign and symptom, and controlling their blood pressure and fetal movements in an outpatient basis. Reliable identification of PD cases before clinical features are evident will also help clinicians focus on the appropriate patients, in which they shall make a targeted ultrasound examination by an expert examiner and clinical follow-up, maturing fetal lungs and procuring fetal and maternal neuroprotection with magnesium sulfate before delivery when indicated, and referring to a tertiary health care hospital in a stable condition when necessary. However, this strategy performs poorly for identifying late-onset PD cases, and it seems reasonable to reassess the angiogenic status in all pregnancies with persistent abnormal UtA Doppler at about 4–5 weeks later (30–33 weeks), as it has been recently proposed [50]. It must be kept in mind that although there is an approximately four-to six-fold higher risk of maternal and perinatal adverse outcomes among early-onset PE cases when compared to late-onset PE, the later cases are more common so that, in absolute terms, the latter accumulate the largest number of complications [51].

(3) *Fetal interest*: PD-related disorders developing before 24 weeks is a very rare condition in which perinatal survival is less than 10% and severe maternal complications are common if PE is present. In these cases, the diagnosis is usually evident, expectant management is not justified and even termination of pregnancy may be offered, so that selection of candidates for intensive surveillance before 24 weeks seems to have little sense [52]. Between 24 and 26 weeks, perinatal survival exceeds 60% if managed in specialized centers, but neonatal complications remain very high. In the presence of IUGR, survival does not reach 50% before 26 weeks [53,54]. Therefore, recommended expectant management at 24–26 weeks for PD-related disorders is a matter of concern, especially if IUGR is detected. From 26 to 34 weeks, perinatal morbidity and mortality decrease as gestational age advances and these are the cases that benefit the most from specialized expectant management to achieve pregnancy prolongation until data of inevitably worsening disease appears. Consequently, we advocate the initiation of intensive surveillance in well-stratified women from approximately 26 weeks in order to provide the maximum benefit for the fetus.

### 3.3. Aid in Early Diagnosis in Symptomatic Patients

The main argument against the use of the angiogenesis-related factors during the second half of pregnancy is the lack of available treatments able to modify the natural history of PD. However, it has been demonstrated that the only modifiable prognostic factor for IPD is the optimization of medical care. This is obvious when it is observed that fetal and maternal morbidity and mortality related to PD are much greater in the developing world, but also in developed countries significantly-diminishing maternal complications from PE has been demonstrated when standardized surveillance was introduced (5.1% *vs.* 0.7%, *p* < 0.001) [55]. Similarly, serial Doppler assessments of IUGR fetuses are useful to determine the time and mode of delivery and thereby to improve the prognosis [4].

The preliminary results of the “PROGNOSIS” study have shed new light on the usefulness of the sFlt-1/PlGF ratio for the triage of women with suspected PE: a single cut-off of 38 can rule out PE (if <38) within 1 week, with a negative predictive value >99%, and rule in PE (if ≥38) within four weeks, with a positive predictive value of nearly 40% [56]. These impressive results have not been previously achieved by any other available test or combination of tests. The negative predictive value of the angiogenesis-related biomarkers could be of great value on arrival for triage of suspected PD-related disorders, by avoiding overtesting and overtreating, including unnecessary admissions and iatrogenic preterm deliveries [57]. Concerning the efficiency of serum biomarkers, a study has been carried out whereby clinical appliance of sFlt-1/PlGF ratio in stratification of patients at risk of PE might reduce costs and use of resources [58].

### 3.4. Aid in Management

sFlt-1/PlGF ratio might also be useful for monitoring already-diagnosed patients, thus providing prognostic information as it correlates with the duration of pregnancy [59]. Diagnosing women before PD complications arise will allow a pondered guidance on pregnancy termination with greater guarantees. At a cut-off of 85, the sFlt-1/PlGF ratio can diagnose early-onset PE with a sensitivity of 89% and a specificity of 97%. In an outstanding study, Rana *et al.* [60] used this cut-off point to predict adverse pregnancy outcomes showing that, in cases evaluated under 34 weeks of gestation, delivery took place in the two weeks following its determination in 86% of patients with a sFlt-1/PlGF ratio ≥85, compared with only 16% of women with a sFlt-1/PlGF <85. In our experience, when the sFlt-1/PlGF ratio is that increased under 32 weeks of gestation there is no hesitation for the diagnosis of PD. Moreover, once PE is diagnosed, we have confirmed an inverse relationship between the sFlt-1/PlGF ratio values and time to delivery: in early-onset PE, pregnancies with values >655 lasted more than 48 h in only 29.4% of cases, and longer than seven days in 5.9% (*vs.* 50% and 30.8% respectively when the value was ≤655 [59].

The great majority of studies carried out until today are based on unique or serial measurements of sFlt-1/PlGF ratio separated by several weeks. However, the analysis of its trend in a short term might add more information on the prognosis of each patient. In a preliminary study it has been noticed that sFlt-1/PlGF ratio values tend to duplicate in 48 h when it lasts less than five days for the need of pregnancy termination because of maternal or fetal complications, underlying the importance of its narrow serial measurement [61].

In summary, the introduction of the sFlt-1/PlGF ratio in the diagnostic algorithm of PE, added to unspecific classical signs and symptoms, may provide better reproducibility and objectivity. Furthermore, they are easy to obtain and usually have an easy interpretation. In this sense, some cautions should be noted, since most of the previous studies have been conducted on selected populations, and the performance of these markers has not been sufficiently studied in special situations, such as multiple gestations [62] or pregnancies affected by congenital infections [63], chromosomal abnormalities [64] or cardiac malformations [65], in which angiogenic markers may have a more complex interpretation. Moreover, as they are based on the pathogenesis of the disease, allow a precise and reliable identification of PD with angiogenic imbalance (whose association to complications is greater than in PD without angiogenic imbalance) [17]. It must be taken into account that angiogenesis-related biomarkers provide surrogate information about placental function, which can be altered in a variety of PD diseases, so that sFlt-1/PlGF ratio may increase to similar levels in IUGR and in PE [16]. However, this does not mean that PD-related disorders without angiogenic disturbance do not need to be carefully monitored, but the risk of complications is significantly lower.

## 4. Proposed Algorithm for the Clinical Integration of the sFlt-1/PLGF Ratio throughout Pregnancy

An adequate identification of pregnant women in which measuring angiogenic biomarkers is the first main challenge for the standard implementation of the sFlt-1/PlGF ratio, since its relatively-elevated cost makes, nowadays, the analysis unfeasible for the general population. A correct categorization of candidates to be applied the sFlt-1/PlGF ratio relies on patients’ stratification of risk for developing PD. The implantation of PD screening in the first trimester should be the elective selection method if any preventive attitude, such as the use of low dose aspirin from the first trimester, is shown to improve obstetric outcomes. Until then, the determination of the mid-trimester UtA Doppler—a much more extended test—could be considered as the cornerstone to identify women in whom the sFlt-1/PlGF can be applied more efficiently.

**Figure 2:**
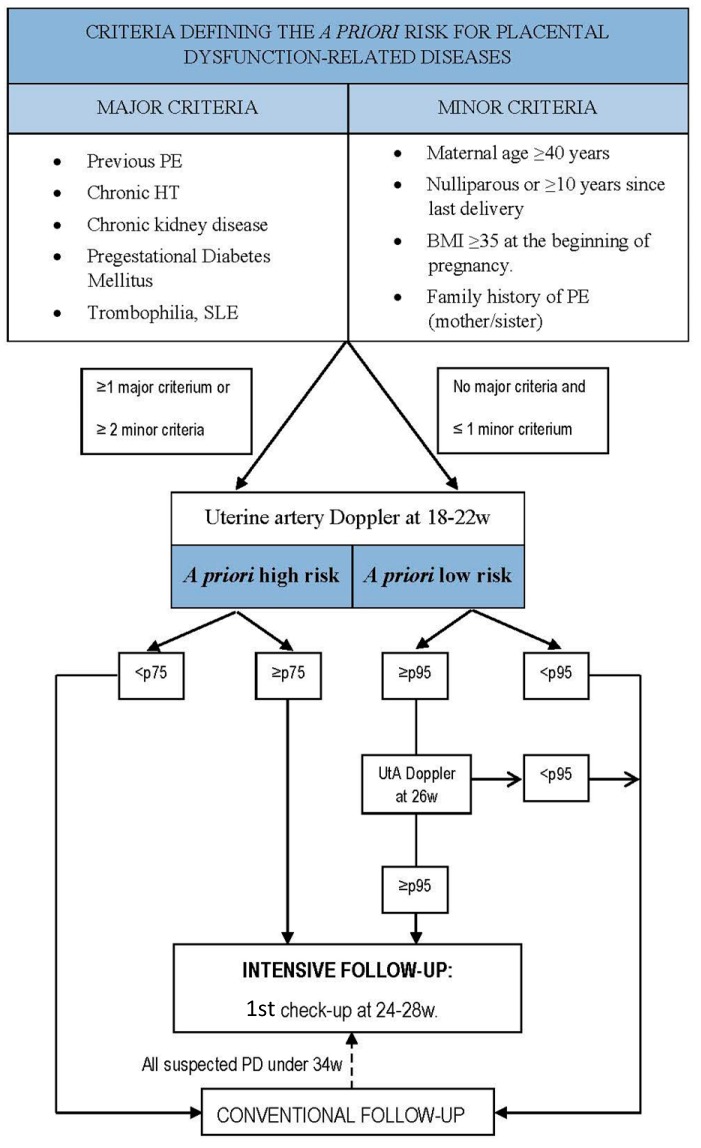
Algorithm for the selection of patients at risk of developing placental dysfunction-related diseases. BMI, body mass index (kg/m^2^); HT, hypertension; p, percentile; PE, preeclampsia; PD, placental dysfunction; UtA, uterine arteries; w, weeks of gestation.

**Figure 3:**
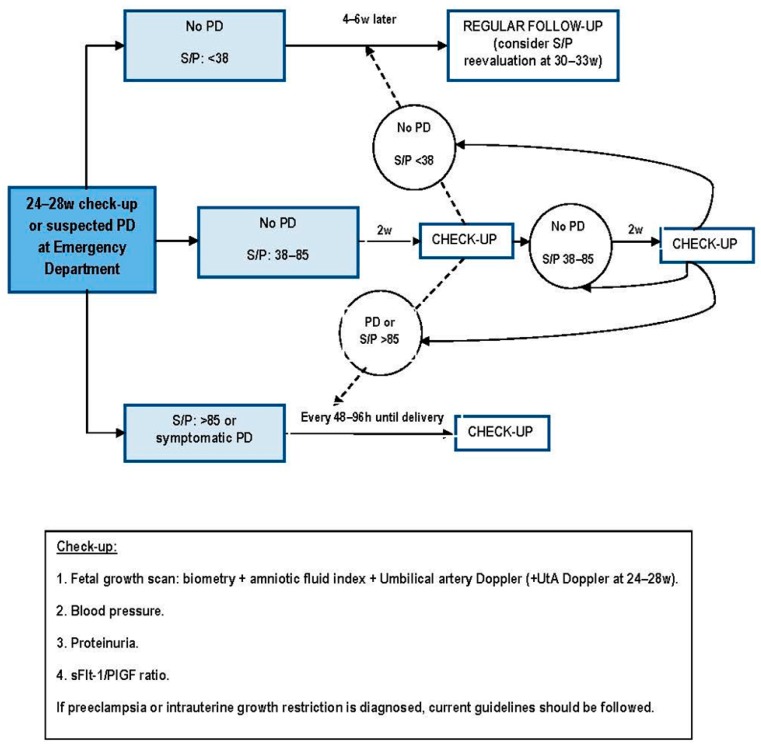
Algorithm for the intensive following-up of patients at risk of developing placental dysfunction-related disorders, including the implementation of angiogenesis-related biomarkers. PD, placental dysfunction; S/P, sFlt-1/PlGF ratio; UtA, uterine arteries; w, weeks of gestation.

As mentioned above, several maternal factors are known to increase the risk of PD. However, they do not all have the same influence, thus allowing categorizing patients as low or high *a priori* risk depending on their personal history (Figure 2). Once patients are properly categorized, we do propose the use of different cut-off points for the UtA Doppler resistances depending on their *a priori* risk, since it performs poorly in high-risk women: ≥75th percentile for women at high risk and ≥95th percentile for women at low risk. We also do propose its re-evaluation at about 26 weeks in women at low *a priori* risk, in order to make an intensive monitoring only in those patients with persisting resistances ≥95th percentile (Figure 3). Patients with suspected early-onset PD in the emergency department are also susceptible to the measurement of the sFlt-1/PlGF ratio, irrespective of their previous UtA Doppler.

Based on this rational use of the sFlt-1/PlGF ratio, a first measurement is made at 24–28 weeks of gestation only in approximately 5% of general pregnant population. Here, three possibilities may exist:

- sFlt-1/PlGF ratio under the “rule out” cut-off point (<38): there is a low risk of developing any early-onset PD, and women can undergo a regular follow-up, tailored to their pregnancy characteristics.
- sFlt-1/PlGF ratio in an intermediate range (38–85): these women are highly likely to develop clinical manifestations of PD within four weeks. A new biomarkers’ determination is needed two weeks later, as this is the lapse considered to be safe of pregnancy complications when sFlt-1/PlGF ratio values are elevated under 85.
- sFlt-1/PlGF ratio upper than the “rule in” cut-off point (>85), or symptomatic PD: those patients should be considered as having a PD, and managed as current guidelines, adding regular reappraisals of the sFlt-1/PlGF ratio every 48–96 h for the support of clinical management (Figure 2). If clinical diagnosis of a PD-related disorder has not been reached, it should be actively pursued.

This innovating strategy for the implementation of angiogenic biomarkers in the clinical routine is being currently applied in our center. Of course, the adopters of these biomarkers should be aware that their use is intended to help clinicians to discriminate the PD-related disorders, but they cannot replace the ordinary clinical antenatal care, especially in cases of late-onset. Our preliminary, but still unpublished, data after analyzing 167 singleton pregnancies fulfilling inclusion criteria for intensive surveillance and with placental insufficiency complications in *n* = 42 (25.1%) [IUGR in *n* = 21 (12.6%), PE *n* = 6 (3.6%) and PE + IUGR *n* = 15 (8.9%)], showed that the area under the ROC curve for the detection of early-onset PE was 0.89 (95% CI: 0.80 to 0.97) and for early-onset IUGR of 0.94 (95% CI: 0.89 to 0.99). For a specificity of 90%, the sensitivity was 81% and 86%, respectively. None of the false-negative cases debuted before 32 weeks. However, in the late phase the performance of the sFlt-1/PlGF measured at 24–28 weeks was much poorer, with an area under the ROC curve for the detection of late-onset PE of 0.64 (95% CI: 0.29 to 0.99) and for late-onset IUGR of 0.67 (95% CI: 0.49 to 0.85). For a specificity of 90%, the sensitivity was 60% and 39%, respectively. These later results highlight the need to reassess the angiogenic balance by 30–33 weeks, and to take into account the less discriminative ability of these biomarkers as the pregnancy progresses.

Recently, this strategy has also been adopted by other large maternity hospitals in Spain. If the results obtained are as good as they seem to be, a widespread use of the angiogenesis-related biomarkers might come in the near future to help improving patients’ risk stratification, diagnostic and management, and reducing iatrogenic attitudes, thus improving maternal and perinatal outcomes in PD.

## 5. Conclusions and Future Perspective

PD-related disorders are leading causes of maternal and perinatal morbidity and mortality as well as iatrogenic prematurity. Current diagnosis of PD is based on nonspecific clinical symptoms and laboratory findings. Recently, angiogenesis-related biomarkers—mainly the sFlt-1/PlGF ratio—have been incorporated in some centers into clinical practice for aid in prediction, early diagnosis, and monitoring the disease course of PD. Current knowledge about the utility of these biomarkers has permitted us to design a strategy for their implementation into the prenatal care. New studies are needed to demonstrate the benefits of the use of the sFlt-1/PlGF ratio in terms of fetal and maternal risks reduction and resource optimization. Novel therapies focused on the removal or in antagonizing sFlt1 are also under investigation and there is a hope that in the near future the complications of PD will no longer be an unpredictable and frightening obstetric complication.
